# First Release of the Bacterial Biobank of the Urban Environment (BBUE)

**DOI:** 10.1128/MRA.01201-18

**Published:** 2018-10-25

**Authors:** Verónica Antelo, Cecilia Salazar, Arací Martínez, Bruno D’Alessandro, Marta Castro, Laura Betancor, María Victoria Barcala, Diana Míguez, Gastón H. Gonnet, Gregorio Iraola

**Affiliations:** a Proyecto Centro de Metagenómica, Institut Pasteur de Montevideo, Montevideo, Uruguay; b Departamento de Bacteriología y Virología, Instituto de Higiene, Universidad de la República, Montevideo, Uruguay; c Laboratorio de Evaluación de la Calidad y Control Ambiental, Intendencia de Montevideo, Montevideo, Uruguay; d Laboratorio Tecnológico del Uruguay (LATU), Montevideo, Uruguay; e Department of Computer Science, ETH Zurich, Zurich, Switzerland; f SIB Swiss Institute of Bioinformatics, Lausanne, Switzerland; g Unidad de Bioinformática, Institut Pasteur de Montevideo, Montevideo, Uruguay; h Centro de Biología Integrativa, Universidad Mayor, Santiago de Chile, Chile; Indiana University Bloomington

## Abstract

Metagenomics is providing a broad overview of bacterial functional diversity; however, culturing and biobanking are still essential for microbiology. Here, we present the Bacterial Biobank of the Urban Environment (BBUE), a sizable culture collection for long-term storage and characterization of the microbiota associated with urban environments relevant for public health.

## ANNOUNCEMENT

Culture-independent approaches like metagenomics are being increasingly applied to characterize the host-associated microbiota and also the diverse microbial communities colonizing natural environments (1, 2), including urban areas, where high concentrations of human-associated bacteria are released into the environment (3). Although these methodologies are allowing us to expand our understanding of the microbial world with unprecedented resolution, traditional microbiology is still fundamental to uncover bacterial ecology, evolution, and virulence. Accordingly, culture collections serve as repositories of microbial diversity and are essential for the long-term availability of relevant strains (4).

Here, we present the first version of the Bacterial Biobank of the Urban Environment (BBUE), a still-in-expansion culture collection from urban waters of Uruguay. Given the increasing awareness about the microbiota that colonizes urban environments (5), we initiated this collection by sampling beaches and sewage pipes due to the role of environmental waters as a vehicle for transmission of clinically relevant bacteria.

Water samples were collected in sterile plastic bottles and processed within the same day. Serial dilutions were prepared using autoclaved distilled water, and 100 ml of each dilution was filtered through 0.45-μm-pore-sized nitrocellulose membrane filters (Sartorius). Membranes were placed upon plates with a modified MacConkey agar (peptone, 20 g/liter; lactose, 10 g/liter; bile salts, 5 g/liters; NaCl, 5 g/liter; and agar, 16 g/liter) and incubated overnight at 35°C under aerobic conditions to look for Gram-negative bacteria. Colonies were picked from the filter and subcultured until pure isolates were obtained, which were finally preserved in 20% glycerol at −80°C. Initial species identification was performed by matrix-assisted laser desorption ionization–time of flight mass spectrometry (MALDI-TOF MS) using a MALDI Biotyper (Bruker Daltonik). Isolate genomes are being sequenced, assembled, and annotated using previously reported approaches (6, 7).

Currently, the collection comprises 232 bacterial strains that are classified into 12 genera. Escherichia coli strains represent the most abundant species (*n =* 96) and were isolated exclusively from sewage water. The genus Pseudomonas comprises 43 strains. Out of them, 36 (84%) are identified to the species level, including Pseudomonas monteilii (*n* = 16), Pseudomonas putida (*n* = 14), Pseudomonas aeruginosa (*n* = 3), and Pseudomonas koreensis (*n* = 2). Strains belonging to the genus Klebsiella include those of Klebsiella pneumoniae (*n* = 38) and Klebsiella oxytoca sp. (*n* = 2). The genus Enterobacter is represented by 18 strains. Out of them, 14 (78%) are identified to the species level, including eight strains of Enterobacter cloacae and six classified as Enterobacter asburiae. The collection also includes members of the genera Raoultella (*n* = 14), Aeromonas (*n* = 2), Citrobacter (*n* = 3), and Stenotrophomonas (*n* = 1) and the species Kluyvera ascorbata (*n* = 1), Proteus mirabilis (*n* = 1), Acinetobacter haemolyticus (*n* = 1), and Staphylococcus capitis (*n* = 2). Metadata are available at https://doi.org/10.6084/m9.figshare.7088639.v2.

Biobanking enables genomic and phenotypic characterization, allowing better understanding of the interplay between relevant traits (such as virulence or antibiotic resistance) of hazardous bacteria and the resident urban microbiota. The Bacterial Biobank of the Urban Environment (BBUE) is a sizable initiative that underpins whole-genome and metagenomic sequencing aiming to integratively assess microbiological risks at the city scale. In the future, we expect to enlarge this collection by culturing isolates from other environments under alternative growing conditions to expand taxonomic and functional diversity.

### Data availability.

Bacterial isolates at the Institut Pasteur Montevideo (Uruguay) are available upon request and can be shipped as a pure bacterial culture inoculated onto solid agar or as a lyophilized culture.
